# Medicinal Plants and Phytochemicals for the Treatment of Pulmonary Hypertension

**DOI:** 10.3389/fphar.2020.00145

**Published:** 2020-03-12

**Authors:** Seyed Vahid Jasemi, Hosna Khazaei, Ina Yosifova Aneva, Mohammad Hosein Farzaei, Javier Echeverría

**Affiliations:** ^1^ Department of Internal Medicine, Kermanshah University of Medical Sciences, Kermanshah, Iran; ^2^ Pharmaceutical Sciences Research Center, Health Institute, Kermanshah University of Medical Sciences, Kermanshah, Iran; ^3^ Institute of Biodiversity and Ecosystem Research, Bulgarian Academy of Sciences, Sofia, Bulgaria; ^4^ Departamento de Ciencias del Ambiente, Facultad de Química y Biología, Universidad de Santiago de Chile, Santiago, Chile

**Keywords:** pulmonary hypertension, medicinal plants, phytochemicals, herbal medicine, phytopharmacological review

## Abstract

**Background:**

Pulmonary hypertension (PH) is a progressive disease that is associated with pulmonary arteries remodeling, right ventricle hypertrophy, right ventricular failure and finally death. The present study aims to review the medicinal plants and phytochemicals used for PH treatment in the period of 1994 – 2019.

**Methods:**

PubMed, Cochrane and Scopus were searched based on pulmonary hypertension, plant and phytochemical keywords from August 23, 2019. All articles that matched the study based on title and abstract were collected, non-English, repetitive and review studies were excluded.

**Results:**

Finally 41 studies remained from a total of 1290. The results show that many chemical treatments considered to this disease are ineffective in the long period because they have a controlling role, not a therapeutic one. On the other hand, plants and phytochemicals could be more effective due to their action on many mechanisms that cause the progression of PH.

**Conclusion:**

Studies have shown that herbs and phytochemicals used to treat PH do their effects from six mechanisms. These mechanisms include antiproliferative, antioxidant, antivascular remodeling, anti-inflammatory, vasodilatory and apoptosis inducing actions. According to the present study, many of these medicinal plants and phytochemicals can have effects that are more therapeutic than chemical drugs if used appropriately.

## Introduction

### A Brief Overview of Lung Circulatory System

The lung is a metabolic organ with unique circulatory system. It has two types of blood circulation: 1) pulmonary circulation that is responsible for gas exchange and 2) bronchial circulation (called pulmonary collateral circulation, systemic circulation) that carries oxygenated blood to the bronchi and trachea, extra pulmonary and intra pulmonary airways, nerves and lymph nodes. Pulmonary arteries have thinner walls without basal tone and with less vascular smooth muscle compared to other arteries. Factors that affect lung circulation are vascular structure, hormonal and neuronal agents and oxygen uptake from the environment. The most important function of the pulmonary circulation is gas exchange to provide blood-containing oxygen for organs consumption. CO_2_-rich blood from the right atrium and right ventricle and the pulmonary artery is transferred to the lung for gas exchange; then oxygen-rich blood is transferred *via* pulmonary vein to the left atrium and left ventricle and then delivered to the body through the aortic artery. Bronchial circulation provides oxygen and nutrients for airway wall and inflammatory cells needed for immune response in airway mucosa, and cleaning the airway from inflammatory mediators and infected particles (Suresh and Shimoda, 2016).

### Pulmonary Hypertension: General Definition and Epidemiology

Pulmonary hypertension (PH) is a disease associated with increase in pulmonary vasculature resistance due to increase in vascular tone and remodeling the structure of pulmonary arteries (PAs). PH occurs predominantly in women (80%), over the mean age of 53 years (Simonneau et al., 2013). It has been reported about 100 million people around the world suffering from PH (Hui-li, 2011). Approximately every untreated patient with PH could survive for about 2.8 years. Some of the PH symptoms include permanent hypoxia, inflammation of lungs, oxidative stress, increase of proliferation of endothelial cells in pulmonary arteries and inhibition of apoptosis, which leads to pulmonary vascular remodeling (Schermuly et al., 2011).

One of the results of PH is an increase in the volume of pumped blood from the heart and increased pulmonary vasoconstriction to compensate the lack of oxygen in the lungs (Reyes et al., 2018); when blood volume increased, it leads to increased right ventricular pressure and right ventricular hypertrophy and finally leads to right ventricular failure (Julian and Mirsalimi, 1992). Increase the pulmonary arterial pressure leads to right ventricle hypertrophy (RV/TV ratios) and increase circulating blood volume of the lungs, compared to normal conditions. Rapid blood flow moving through the lungs vasculature causes disturbance in O_2_ and CO_2_ exchange (Powell et al., 1985).

The most important indexes for PH are fatigue and dyspnea during walking or moderate physical activity. The inability to do daily functions in these people leads to decrease their quality of life. PH is difficult to diagnose because of its similarities of symptoms to the other cardiopulmonary disease; as a result, the disease progresses and leads to increased mortality (Benza et al., 2010). The final diagnosis is through clinical assays for the detection of hemodynamic abnormalities. In most patients that suffering from PH, right ventricular hypertrophy is seen in the electrocardiogram and pulmonary function tests, confirm a decrease in lung capacity (Galie et al., 2009).

### Classification and Etiology

PH is classified into five groups based on its etiology: i) Hypertension in the pulmonary arteries that lead to pulmonary arterial hypertension (PAH); ii) Left-sided heart failure that leads to PH; iii) Hypoxic pulmonary vasoconstriction that leads to PH; iv) High pressure in the blood vessels of the lungs that leads to chronic thromboembolic pulmonary hypertension (CTEPH), and v) Idiopathic PH (unknown reason).

Many studies suggested the effect of various factors such as high altitude, low temperatures, intense light, air pollution, type of nutrition, oxidative stress and gene mutations on the progression of PH (Baghbanzadeh and Decuypere, 2008). Based on these studies, the use of antioxidant compounds could be effective in treatment and control of PH (Wong et al., 2013).

Approximately 50% of patients suffer from idiopathic PAH. Diverse mutations are responsible for PH including ALK-I, BMPR2, endoglin, and CAV-I (Simonneau et al., 2013) too. Studies have proven the effects of nutrition on the progression of PH, such as calorie intake, amount of minerals and pure proteins (Khajali and Fahimi, 2010). The cellular and molecular reasons presented bellow could explain occurrence of PH.

### Cellular/Molecular Changes during PH Progression

Some of the most important cellular and molecular changes during PH include smooth muscle cells remodeling, uncontrolled proliferation of endothelial cells, inflammation and the presence of proinflammatory cells, platelet aggregation and thrombosis (Humbert et al., 2004).

#### Smooth Muscle Cells and Remodeling

During the progression of PH, smooth muscle cells are remodeling to small peripheral, pulmonary arteries inside the respiratory acinus are observed; in other words, increase of smooth muscle cells of the distal pulmonary arteries, also known as pathological remodeling. The cellular process of this mechanism is incompletely understood. Anyway, during the process, a layer of myofibroblasts and extracellular matrix are created between the endothelium and internal elastic lamina (Stenmark et al., 2002). The first mechanism of fibroblast to media migration is increased expression of matrix metalloproteinases (MMP2 and MMP9). In summary, all the mechanisms involved in these processes lead to increase the thickness and obstruction of the pulmonary arteries (Davie et al., 2004).

#### Uncontrolled Proliferation of Endothelial Cells

All of the reasons for abnormal proliferation of endothelial cells occur on genetic background, but some of them are due to oxidative stress, hypoxia conditions, inflammation, allergy of drugs or toxins. It has been proven that patients with idiopathic PAH have defects in growth-suppressive genes such as BAX and TGF-β (Yeager et al., 2001). Approximately 30% of lesions, a mutation in the TGF-βR2 gene and 90% inhibition of expression of TGF-βR2 protein were observed (Yeager et al., 2001). Studies show that vasculotropic viruses such as human herpesvirus 8 infection could be effective in idiopathic PAH by changing the signaling pathways involved in endothelial cells proliferation (Cool et al., 2003). TGF-β1 is an important factor in the process of PH that is associated with endothelial injury and vascular remodeling. Studies suggest that TGF-β1 stimulates the production of ET-1 and that leads to increase of the smooth muscle cells proliferation (Star et al., 2009). Also, TGF-β1 initiates endothelium-smooth muscle transformation (Masszi et al., 2004).

#### Inflammation and Proinflammatory Cells

Inflammatory factors are the important ones in the progression of PH of human and animal models (Dorfmüller et al., 2003). In some patients, the suppression of the immune system shows positive results in reduction of the inflammation (Dorfmüller et al., 2003). Also, it has been reported that there are elevation of proinflammatory cytokines (IL-1 and IL-6) levels in patients with idiopathic PH. Histological studies of the lungs confirmed the presence of macrophages and lymphocytes and increased expression of chemokines CCL5 and fractalkine in severe PH (Balabanian et al., 2002).

#### Platelets Aggregation and Thrombosis

A notable mechanism in PH progression is thrombosis lesions due to platelet aggregation. This pathway can be initiated by endothelial cells, inflammatory mediators or by the platelets. Blood clot formation in PH patients is characterized by increase of fibrinopeptide A- and D-dimers levels in the plasma. Current studies show that stress itself and pulmonary vascular injury can initiate the process. Other evidence suggests that vascular abnormalities in PH leads to release of procoagulant, vasoactive, and mitogenic mediators by platelets. Some contributing factors in pulmonary vasoconstriction like serotonin, TXA2, TGF-β, AGEPC, PDGF and VPF can be store and release by platelet (Herve et al., 2001).

### Animal Models

Briefly, animal models used in PH studies are divided into several groups according to the type of induction: hypoxia induces PH in mice and rats (Rabinovitch et al., 1979) and various types of cattle with genetic background (Shirley et al., 2008); fawn-hooded rat (FHR) that spontaneously develop PH in mild hypoxia conditions (Nagaoka et al., 2001); monocrotaline that induces PH by increased inflammation in mice and rats (Kay et al., 1967), genetically pulmonary hypertensive rats and mice that develop PH by loss-of-function mutation of *BMPR2* gene in smooth muscle (Beppu et al., 2004).

### Restrictions of Current Treatments of PH

Some of the commonly used drugs for the treatment of PH are phosphodiesterase-5 inhibitors such as sildenafil (Pepke-Zaba et al., 2008), prostacyclin analogs such as epoprostenol (Sitbon et al., 2002), and antagonists of endothelin receptor such as bosentan (McLaughlin et al., 2006). Some of the effective oral medications include anticlotting drugs, urine-enhancing drugs, oxygen-enhancing drugs, vasorelaxant drugs and calcium channel inhibitors. These treatments improve the quality of life during the first 3 years, but there is no cure for improvement of patient's quality of life for 5 years (Waxman and Zamanian, 2013). Also, these cures have not been completely successful because of side effects or incomplete efficacy (Humbert et al., 2004). Due to the important role of ROS and oxidative stress in PH progression, the intake of antioxidant compounds, which aims to reduce oxidative damage, is one of the notable issues in the treatment of PH (Wong et al., 2013).

## Methods

### Study Design

Electronic databases used in this review study were PubMed, Scopus, and Cochrane library. The keywords used were ‘pulmonary hypertension' in the title/abstract and ‘plant', ‘herb', ‘phytochemical' in the whole text. The period of articles reviewed is between 1979 and 2019. The primary search was performed by two researchers separately, and unrelated articles were separated based on their title and abstract. In the present study, both clinical and animal studies were included in the search but the number of clinical trials were very limited, and therefore the number of the results from the Cochrane library are fewer than these from Scopus and PubMed. Unrelated articles based on title and abstract, non-English and duplicate ones were excluded in the first step, then remaining articles were reviewed in full text. In the second step, several articles were excluded based on the full text. Study design diagram is available in the results section.

### Data Extraction

All data were extracted and studied by two researchers and were summarized in **Tables 1** and **2**. Abstract information of articles, including the name of the plant and the category of phytochemicals, effective dose, the type of PH model, and outcomes of the treatments, were categorized in **Tables 1** and **2**. The ↑ and ↓ signs show significant increase and significant decrease respectively of the evaluated factors during the mentioned studies.

**Table 1:** Medicinal plants used for Pulmonary Hypertension treatment.

Medicinal plants (Scientific Name)	Effective dose	Extract/Part	Model	Animal/Cell	Outcome	References
*Allium macrostemon* Bunge	0.03% mix in food	Volatile oil/Bulb	_	*In vitro*-PAECs	↑eNOS, ↑Ca^2+^ influx in PAECs	Han et al., 2017
*Allium sativum* L.	100 mg/kg	Aqueous extract/Garlic powder	Hypoxia	*In vivo*-rat	↑NO, ↓PH	Fallon et al., 1998
*Allium ursinum* L.	2% mix in food	ND/Leaf	MCT	*In vivo*-rat	↓RV/(LV + S), ↓MWT %, ↑PDE5A, ↑TAPSE	Bombicz et al., 2017
*Crataegus rhipidophylla* Gand. (syn. *Crataegus oxyacantha*)	0.2 mL/L drinking water	Flavonoid enrich extract/ND	High altitude	*In vivo*-broiler chicken	↑BW, ↓PBP, ↑NO, ↓MDA, ↓hematocrit, ↓H:L ratio, ↓RVH	Ahmadipour et al., 2019
*Crataegus rhipidophylla* Gand. (syn. *Crataegus oxyacantha*)	0.2 mL/L drinking water	Extract mix in drinking water	High altitude	*In vivo*-broiler chicken	↓AST, ↓ALT, ↓ALP, ↑Albumin, ↑Globulin,↑Feed intake, ↓RV : TV ratio	Ahmadipour et al., 2017
*Eulophia macrobulbon* (E.C.Parish & Rchb.f.) Hook.f.	15 and 450 mg/kg	Ethanolic extract/tuber	MCT	*In vivo*-rat	↑PA relaxation, ↓(RV/LV+S), ↓RVH	Wisutthathum et al., 2018
*Kelussia odoratissima* Mozzaf	0.75% mix in food	Mix in food/leaves and shoots	High altitude	*In vivo*-broiler chicken	↑NO, ↓MDA, ↓H:L ratio, ↓Hematocrit, ↓ET-1, ↑SOD, ↑iNOS	Ahmadipour et al., 2015
*Mimosa pigra* L.	*In vivo*: 400 mg/kg *In vitro*: 0.01-1 mg/mL	Hydro methanolic extract/leaf	Hypoxia	*In vivo*-rat/*In vitro*-HPVECs	↑NO, ↓PAP, ↓RV/(LV+S), ↓p38, ↓^.^O^-^_2,_ ↓TNF-α	Rakotomalala et al., 2013
*Moringa oleifera* Lam.	4.5 mg/kg	Ethanol extract/leaves	MCT	*In vivo*-rat	↓PABP, ↑SOD	Chen et al., 2012
*Rhodiola tangutica* (Maxim.) S.H.Fu (syn. *Sedum algidum* var. *tanguticum* Maxim.)	250 mg/kg	Bioactive enrich fraction	Hypoxia	*In vivo*-rat	↓mPAP, ↓RVH, ↓PCNA, ↓cyclin D1, ↓CDK4, ↑p27Kip1, ↓pulmonary small artery wall thickness	Nan et al., 2018
*Salvia miltiorrhiza* Bunge	14 g/kg	Aqueous extract/Root	MCT	*In vivo*-rat	↓PAP, ↓ET-1, ↓TXA2, ↑NO, ↑Prostacyclin, ↓TGF β1, ↓mPAP, ↓RVSP	Wang Y. et al., 2015
*Salvia miltiorrhiza* Bunge	ND	ND	Hypoxia	*In vivo-*rat	↓Thickening in the SPAs, ↑HO-1, ↑NO, ↑iNOS, ↓eNOS	Chen et al., 2003
*Salvia miltiorrhiza* Bunge	0.33 mL/100 g BW	ND	Hypoxia	*In vivo*-rat	↓RVSP, ↓RVHI	Si-chuan et al., 1994
*Securigera securidaca* (L.) Degen & Dörfl.	4 g/kg	Supplementation of diet/seed	High altitude	*In vivo*-broiler chicken	↑NO, ↓MDA, ↓T, R, S ECG waves, ↓RVH, ↑iNOS, ↑CAT, ↓ET-1	Ahmadipour, 2018
*Terminalia arjuna* (Roxb. ex DC.) Wight & Arn.	250 mg/kg	Aqueous extract/Stem bark	MCT	*In vivo*-rat	↓SOD, ↑TBARS, ↑CAT, ↓NOX1, ↓BCL2/BAX ratio, ↓%MWT	Meghwani et al., 2017
*Trifolium pretense* L.	20 mg/kg	Isoflavones	Low temperature (12 ± 2°C)	*In vivo*-broiler chicken	↓PAP, ↓ET-1, ↑iNOS	Jiang and Yang, 2016
*Withania somnifera* (L.) Dunal	100 mg/kg	Hydro-alcoholic extract/root	MCT	*In vivo*-rat	↓RVP, ↓RVH, ↓ROS, ↓PCNA, ↑procaspase-3, ↑IL-10, ↓TNF-α, ↓NF kB, ↓HIF-1α, ↑eNOS	Kaur et al., 2015

**Table 2:** Phytochemicals used for Pulmonary Hypertension treatment.

Phytochemical	Effective dose	Model	Animal/cell	Outcome	References
Apiin	5 mM/kg	Hypoxia	*In vivo*-dog	↓PAP, ↓Aortic pressure	Occhiuto and Limardi, 1994
Apple polyphenol	20 mg/kg	Hypoxia	*In vivo*-rat/*In vitro*-PASMCs	↓mPAP, ↓PVR, ↓Cytosolic Ca^2+^ in PASMC, ↓Caspase-3, ↓iNOS ↑NO, ↑eNOS	Hua et al., 2018
Asiaticoside	50 mg/kg	Hypoxia	*In vivo*-rat/*In vitro*-HPAECs	↑NO, ↓ET-1, ↑cGMP, ↑phosphorylation of serine/threonine- protein kinase/eNOS, ↓RVH,↓ mPAP, ↑PI3K/Akt/eNOS pathway	Wang et al., 2018
Asiaticoside	*In vivo*: 50 mg/kg *In vitro*: 100 μg/mL	Hypoxia	*In vivo*-rat/*In vitro*-PASMCs	↓mPAP, ↓RVH, ↓TGF-β1, ↓TGF-βR1, ↓TGF-βR2, ↓Smad2/3	Wang X. B. et al., 2015
Astragalus Polysaccharide	200 mg/kg	MCT	*In vivo*-rat	↓mPAP, ↓PVR, ↓RVH, ↑eNOS, ↑NO, ↓IL-1β, ↓IL-6, ↓TNF-α, ↓pho-IκBα	Yuan et al., 2017
Baicalin	30 mg/kg	Hypoxia	*In vivo*-rat	↑ADAMTS-1, ↓Type I collagen, ↓RV/(LV + S)%, ↓ mSAP, ↓mPAP	Liu et al., 2015
Baicalein	10 mg/kg	MCT	*In vivo*-rat	↓RVSP, ↓RVH, ↓ET-1, ↓ET_A_R, ↑SOD, ↓Akt/ERK1/2/GSK3β/β-catenin, ↑eNOS, ↓vWF	Hsu et al., 2018
Baicalin	40 μg/L	TGF-β1	*In vitro*-HPASMCs	↓TGF-β1, ↓HPASMCs, ↓HIF-1α, ↓AhR	Huang et al., 2014
Berberine	100 mg/kg	Hypoxia	*In vivo*-rat/*In vitro*-PASMCs	↓proliferation and migration of PASMCs, ↓RV/(lV + S), ↓Medial wall thickness, ↓PP2Ac	Luo et al., 2018
Carvacrol	*In vivo*: 100 mg/kg *In vitro*: 600 μM	Hypoxia	*In vivo*-rat/*In vitro*-PASMCs	↓RV/LV+S, ↓MDA, ↑SOD, ↑GSH, ↓Bcl-2, ↓Procaspase-3, ↑Caspase-3, Inhibit the ERK1/2 and PI3K/Akt pathway	Zhang et al., 2016
Genistein	1 mg/kg	MCT	*In vivo*-rat/*In vitro*-HPASMC and NRVM	↓RVP, ↑VEF, ↓RVEF, ↑ERβ ↓HPASMCs, Improvements in cardiopulmonary function, ↓RHF	Matori et al., 2012
Ginsenoside Rb1	*In vitro*: 10 μg	Hypoxia and MCT	*In vivo*-rat/*In vitro* PASMCs	↓ET-1, ↓SOCE, ↓RV(LV+S), ↑Relaxation	Wang R. X. et al., 2015
Isorhynchophyl line (IRN)	*In vivo*: food containing 0.1% IRN *In vitro*: 25 μM	MCT	*In vivo*-rat/*In vitro*-PASMCs	↓Cyclin D1, ↓CDK6, ↑p27Kip1, ↓ERK1/2, ↓STAT3, ↓Akt/GSK3β, ↓RVP, ↓RVH	Guo et al., 2014
Luteolin	5 mM/kg	Hypoxia	*In vivo*-dog	↓PAP, ↓Aortic pressure	Occhiuto and Limardi, 1994
Magnesium lithospermate B	*In vivo*: 15 mg/kg *In vitro*: 20 μM	Hypoxia	*In vivo*-rat/*In vitro*-PASMCs	↓RVSP, ↓NOX2, ↓NOX4, ↓ERK, ↓ROS, ↓H_2_O_2_, ↓OPN, ↓Cyclin D1, ↑α-SMA, ↑SM22α	Li et al., 2019
Nobiletin	50 mg/kg	MCT	*In vivo*-rat/*In vitro*-PASMCs	↓RVSP, ↓RVH, ↓RV/(LV+S) ↓Src/STAT3, ↓PDGF-BB, ↓Src/STAT3	Cheng et al., 2017
Oxymatrine	*In vivo*: 50 mg/kg *In vitro*: 0.2 mg/mL	MCT and hypoxia	*In vivo-*rat*/In vitro-*PASMCs	↓RVSP, ↓RV/[LV+S], ↓Cell number, ↓MCP-1, ↓IL-6, ↓SDF-1, ↓TGF-β, ↓VEGF, ↓ICAM-1, ↓VCAM-1, ↑SOD, ↑HO-1, ↑GSH, ↑Nrf2	Zhang et al., 2014
*Panax notoginseng* Saponins	50 mg/kg	Hypoxia	*In vivo*-rat	↓PAP, ↓RV/(LV+S), ↓p-p38MAPK, ↓p38MAPK	Zhao et al., 2015
Polydatin	20 mg/kg	Hypoxia	*In vivo*-rat	↓mPAP, ↓mCAP, ↓RVH, ↓Ang II, ↓ET, ↑NO	Miao et al., 2012
Punicalagin	45 mg/kg	Hypoxia	*In vivo*-rat	↓RVH, ↓mPAP, ↓RV/(LV+S), ↓MMP-9, ↓HIF-1α, ↓NF-kB, ↓TNF-α, ↓VEGFA, ↑SOD, ↑cGMP, ↑NO	Shao et al., 2016
Quercetin	*In vivo*: 100 mg/kg *In vitro*: 60 μM	Hypoxia	*In vivo*-rat/*In vitro*-PASMCs	↓PAH, ↓RVH, ↓Cell migration, ↑cyclin D1, ↓cyclin B1, ↓Cdc2, ↓MMP2, ↓MMP9, ↓CXCR4, ↓Integrin β1, ↓integrin α5, ↓TrkA/AKT signaling pathway, ↑BAX, ↓BCL2	He et al., 2015
Quercetin	100 mg/kg	MCT	*In vivo*-rat/*In vitro*-PASMCs	↓mPAP, ↓RVH, ↓PCNA, ↓WT	Gao H. et al., 2012
Resveratrol	100 μM/L	Hypoxia	*In vitro*-PASMCs	Inhibiting the PI3K/AKT signaling pathway, ↓p-Akt, ↓MMP-2 and MMP-9	Guan et al., 2017
Resveratrol	*In vivo*: 40mg/kg In vitro: 40 μM	Hypoxia	*In vivo*-rat/*In vitro* PASMCs	↓RVSP, ↓HIF-1α, ↓ROS, ↓PH, ↓IL-6, ↓IL-1β, ↓TNF-α, ↓NF-κB ↑Trx-1, ↑ Nrf-2	Xu et al., 2016
Resveratrol and Trimethoxystilbene	Resveratrol: 200 μM Trimethoxystilbene: 20 μM	TNF-α	*In vitro*-PASMCs	↓PASMCs, TMS was more potent compared with RES	Gao G. et al., 2012
Rhoifolin	5 mM/kg	Hypoxia	*In vivo*-dog	↓Cardiac output, ↓Aortic pressure	Occhiuto and Limardi, 1994
Salidroside	*In vivo*: 32 mg/kg *In vitro*: 800 μM/L	Hypoxia	*In vivo*-rat/*In vitro* PASMCs	↓RVH, ↓BCl2, ↑Caspase-3, ↑BAX, ↑A_2a_R	Huang et al., 2015

## Results

### Study Selection and Characteristics

**Figure 1:**
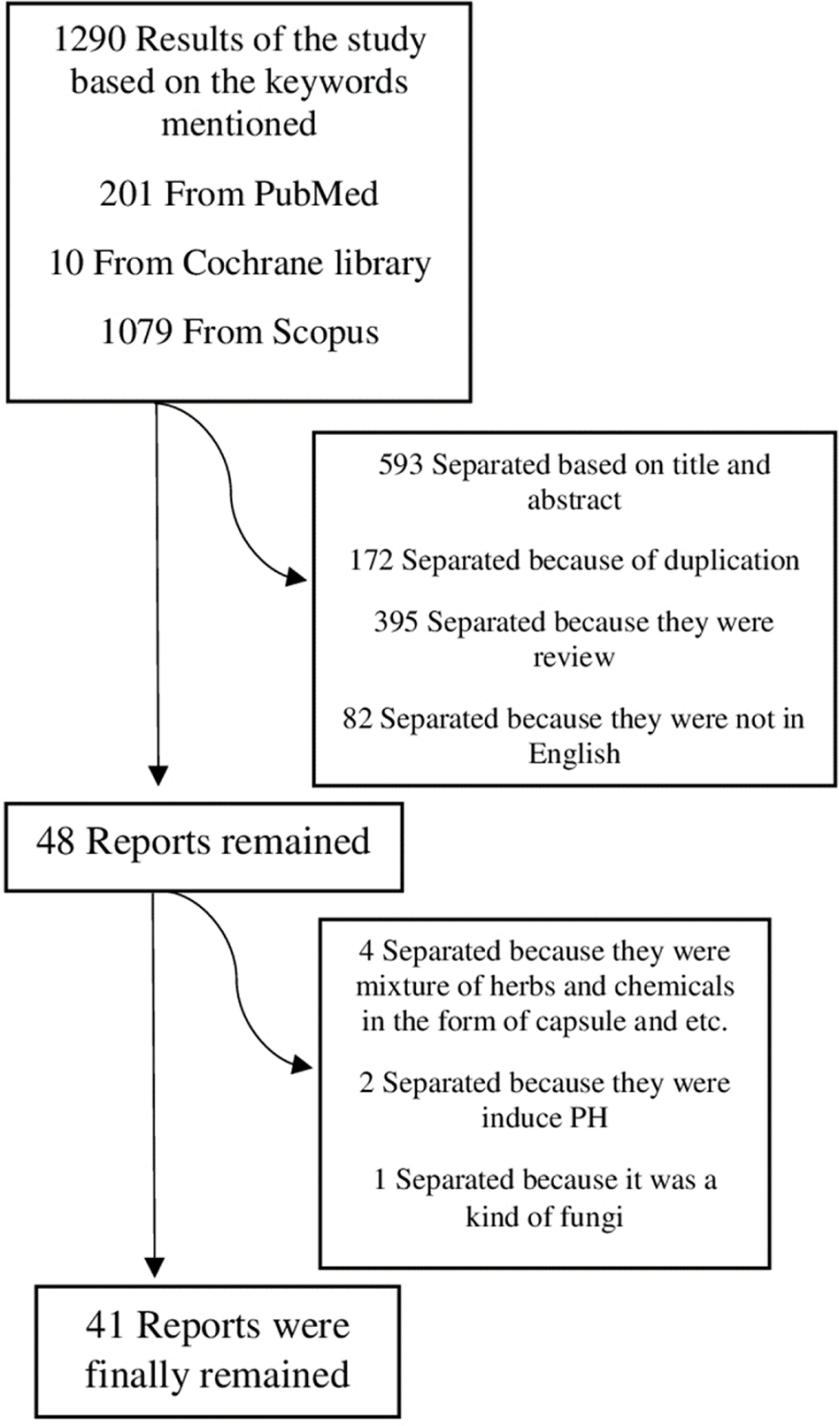
Study design and extraction.

From 1290 studies, 172 studies were separated because of duplication; 395 studies were separated because they were reviews; 593 studies were separated based on their title and abstract; 82 studies were separated because they had not full text in English. From 48 remaining reports, 4 studies were separated because they were mixture of herbs and chemical compounds in the form of capsule; 2 studies were separated because they were induced PH and 1 study was separated because it was for fungus species (not plant). **Figure 1** shows the method of the study and criteria for article separation. The general information of the articles were summarized in **Table 1** and **2**.

#### Medicinal Plants used for PH Treatment

##### Allium sativum *L.*

*Allium sativum* L. (Garlic) is a herbal remedy used in traditional medicine for treatment of a wide range of diseases. The main constituents of *A. sativum* that are responsible for its medicinal actions are sulfur-containing compounds including crysteine sulfoxides. Alliin is part of this group, which could be transformed into thiosulfinates (Reuter et al., 1996; Horníčková et al., 2010).

Some of the therapeutic actions of *A. sativum* such as lowering blood glucose levels, reducing oxidative stress, anticancer, reducing inflammation, immune system enhancer, anti-infectious diseases and cardiovascular protective were demonstrated (Lanzotti et al., 2014). There is evidence suggesting that *A. sativum* could relax vascular smooth muscles, to increase the activity of eNOS, and to hyperpolarize the smooth muscle cell membrane. Fallon et al. (1998) demonstrated that aqueous extract of *A. sativum* powder could prevent the progression of PH induced by hypoxia in rats due to its vasorelaxant activity.

##### Allium macrostemon *Bunge*

*Allium macrostemon* Bunge is a member of Amaryllidaceae family and used in traditional Chinese medicine with many pharmacological activities: antitumor, antioxidant, antiasthmatic (due to its ability to relax the bronchial smooth muscle, reducing blood perfusion and the mean PAs pressure), enhancing immune system (Tan et al., 2012). Dimethyl disulfide (DMDS) is the main constituent of *A. macrostemon* (34.93%) that possesses many biological activities such as regulation of melanin formation, anti-inflammatory and anti-hypertensive, and therefore it is used for treatment of myocardial ischemia (Chu et al., 2017).

The study of Han et al. (2017) demonstrated that use of volatile oil of *A. macrostemon* in PAECs causes increase eNOS expression and serine 1177 phosphorylation. Serine 1177 is a residue for phosphorylation of eNOS in PKA, Akt, and Calmodulin processes (Fleming, 2010). Also, volatile oil of *A. macrostemon* induces relaxation in PAs by activation of intracellular Ca^2+^/PKA/eNOS signaling pathway. Therefore, volatile oil of *A. macrostemon* and its active compound DMDS can improve PH by activation Ca^2+^/PKA/eNOS signaling pathway in PAECs (Han et al., 2017).

##### Allium ursinum *L.*

*Allium ursinum* L. (Wild garlic or bear's garlic), a member of Amaryllidaceae family, is a native plant of Eurasia. It is widely used in traditional medicine (Sobolewska et al., 2015). *A. ursinum* produce a variety of chemical structures responsible for its biological activities. One of the main compounds are flavonoid glycosides and sulfur constituents responsible for its garlic-like fragrance. Some of the *A. ursinum* compounds like galactolipids and phytosterols are species-specific (Oszmianski et al., 2012). Some of the most important pharmacological activities of *A. ursinum* are antiaggregatory effects on human platelets, inhibition effect on 5-lipoxygenase (5-LOX) and prostaglandin-endoperoxide synthase (PTGS) enzymes *in vitro*, high antioxidant activity and inhibition of *in vitro* cholesterol biosynthesis. Its action is also associated with a decrease in blood pressure and phosphodiesterase enzyme (PDE 5A) inhibition. The saponins and flavonoids found in *A. ursinum* could inhibit PDE5A activity (Lines and Ono, 2006). Upregulation of PDE5 has been reported in PH (Kass et al., 2007). The treatment of pulmonary hypertensive rats with *A. ursinum* reverses RVH and the pulmonary vascular remodeling evaluated through the increasing of RV/(LV+S) ratio and the pulmonary arterial wall thickness respectively compared to untreated pulmonary hypertensive rats (Bombicz et al., 2017).

##### Crataegus rhipidophylla *Gand.*

*Crataegus rhipidophylla* Gand. (syn. *Crataegus oxyacantha* L.; Hawthorn) is a member of Rosaceae family and it is native to Europe, Africa and Asia (Chang et al., 2002). Some of the bioactive compounds found in *C. rhipidophylla* (fruits, leaves, and flowers) are epicatechin, hyperoside, and chlorogenic acid. Its extracts have many pharmacological activities such as neuroprotective, hepatoprotective, cardioprotective, nephroprotective (Salehi et al., 2009).

Flavonoid extracts of *C. rhipidophylla* cause improvement in body weight gain, reduction of heterophil to lymphocyte ratio and an increase of NO levels on PH induced by high altitude in broiler chickens (Ahmadipour et al., 2019). Treatment of broiler chickens with these extracts could overexpress the iNOS and SOD-1, and reduce the expression of ET-1 in the heart tissue.

Polyphenols (mainly flavonoids) and oligometric proanthocyanidins (OPCs) compounds of *C. rhipidophylla* are responsible for some of these activities, such as antioxidant, anti-anxiety, anti-cancer and growth inducer. (Surai, 2014).

Treatment with *C. rhipidophylla* extract mix in drinking water of broiler chickens with pulmonary hypertension syndrome (PHS) induced by high altitude suggested that the extract cause an increase in albumin, globulin, food intake and body weight and a decrease in AST, ALT, ALP, and RV : TV ratio (Ahmadipour et al., 2017). ALP is one of the serum's enzymes which is a marker of liver tissue damage and the two liver enzymes, AST and ALT, increase their concentrations in oxidative stress conditions and hypoxic state before PHS. These liver enzymes are sensitive markers for measuring oxidative stress, which is an important factor during PHS progression (Fathi et al., 2011). Therefore, the decrease in ALP, ALT and AST levels show reduction of tissue damage due to oxidative stress and that could lead to inhibition of PHS progression (Ahmadipour et al., 2017).

##### Eulophia macrobulbon *(E.C.Parish & Rchb.f.) Hook.f.*

*Eulophia macrobulbon* (E.C. Parish & Rchb. f.) Hook.f. (Corduroy orchid) is a member of Orchidaceae family. Orchids are commonly used in traditional medicine in Asia, Europe and America. Their pharmacological activities include the treatment of pulmonary, autoimmune and inflammatory diseases, cancer, and diabetes (Narkhede et al., 2016). The ethanolic extract of *E. macrobulbon* contains flavonoids, terpenoids and phosphodiesterase type 5 (PDE5) inhibitor, so it could be a pulmonary vasodilator (Temkitthawon et al., 2017).

Wisutthathum et al. (2018) during a study on MCT induced PH in rats, demonstrated that the treatment with ethanolic extract of *E. macrobulbon* inhibits CaCl_2_-induced contraction in PA rings *in vitro* and reduces (RV/LV+S) ratio. Stilbenes and their derivatives found in *E. macrobulbon* extracts are responsible for inducing relaxation in coronary arteries in hypercholesterolemic pigs (Vilahur et al., 2015). Therefore, it has been suggested that *E. macrobulbon* extract could induce relaxation in PAs (like coronary arteries) and modulate PH (Wisutthathum et al., 2018).

##### Kelussia odoratissima *Mozzaf*

*Kelussia odoratissima* Mozzaf. (Wild celery) is a member of Umbelliferae family, that grows at high altitudes (more than 2000 m a.s.l.), mainly in Iran. It is commonly used in Iranian traditional medicine for hypertension and inflammation treatments. Active compounds of its essential oil are phthalides, (*Z*)-ligustilide and terpenes (Shojaei et al., 2011). The alcoholic extract includes components with high antioxidant activity such as flavonoids and polyphenols (Pirbalouti et al., 2013).

Results of the study of Ahmadipour et al. (2015) on broiler chickens with PH shows improvement in body weight gain, an increase in NO levels and heterophil/lymphocyte ratio and a decrease in MDA levels in serum. In the broiler chickens overexpression of SOD1 and iNOS and suppression of the expression of ET-1 gene in the heart tissue were observed due to treatment with *K. odoratissima*. Thus, this herb could improve the outcomes of PH in broiler chickens.

##### Mimosa pigra *L.*

*Mimosa pigra* L. (giant sensitive tree) is a member of Fabaceae family, popular with its therapeutic application in the countries of Africa, America and Asia. It is used for treatment of many diseases such as cardiovascular diseases, digestive disorders and infections (Rosado-Vallado et al., 2000).

Rakotomalala et al. (2013) in a study on therapeutic potential of *M. pigra* against hypoxia-induced PH in rats, indicated that this plant can increase NO production, reduce PAP and RV/(LV+S) and p38 MAPK expression and phosphorylation in lung tissue. P38 MAPK activated in pulmonary hypertensive rats (Welsh et al., 2005). The results of these studies suggested that *M. pigra* has ameliorative effect on PH outcomes.

##### Moringa oleifera *Lam.*

*Moringa oleifera* Lam. (Drumstick tree) belongs to the Moringaceae family and it is widely cultivated in tropical and subtropical areas. It is native to Northwestern India. Some therapeutic applications of the herb are associated with a decrease in blood pressure, reduction of cholesterol levels, with its high antioxidant, hypoglycemic, anti-ulcer and anticancer activities (Stohs and Hartman, 2015). Nitryl glycosides compounds of *M. oleifera* leaves show hypotensive and antioxidant activities (Gupta et al., 2007). Other studies show that *M. oleifera* can increase the GSH levels in liver tissue (Fakurazi et al., 2008).

Chen et al. (2012) found an increase in SOD levels and a decrease in pulmonary pressure and in pulmonary arterial wall thickness due to treatment with *M. oleifera* in MCT induced PH in rats. It has been demonstrated that niazirin and thiocarbamates (two of the active compounds of *M. oleifera*) have antihypertensive effects on spontaneous hypertension in rats (Faizi et al., 1998). So it could be considered as an useful treatment for PH.

##### Rhodiola tangutica *(Maxim.) S.H.Fu*

*Rhodiola tangutica* (Maxim.) S.H.Fu (syn. *Sedum algidum* var. *tanguticum* Maxim.) (Rhodiola, Golden root) is an important herb used in traditional medicine in Qinghai and Tibetan. It is used for treatment of many diseases especially to prevent colds caused by high altitude (Li et al., 2009). Some of the biologically active substances of enrich fraction of *R. tangutica* are phenylethanols, flavonoids, terpenoids (Nan et al., 2018).

Nan et al. (2018) during a study on hypoxia-induced PH in rats marked that the use of bioactive enriched fraction of *R. tangutica* can decrease mPAP, RV/BW, RV/LV+S, hematocrit and p27Kip1 protein expression and therefore could increase protein expression of PCNA, CDK4, and Cyclin D. These three proteins have key roles in cell cycle switching of G0/G1 phase to S phase. P27kip1 is a CDK inhibitor. Therefore, regulation of these factors can inhibit cell proliferation and following that could inhibit medial vessel thickness, which is a factor for structural changes in pulmonary hypertension.

Vasorelaxant activity of *R. tangutica* was confirmed by *in vitro* study on isometric tension changes induced by a force transducer in rat pulmonary artery (Li et al., 2016). Therefore, according to these studies *R. tangutica* could be considered as an effective treatment for PH.

##### Salvia miltiorrhiza *Bunge*

*Salvia miltiorrhiza* Bunge (Red sage, Danshen) is a member of Lamiaceae family and it is highly valued for its roots. In traditional Chinese medicine, it is used to increase blood flow and cooling the blood in abscess treatment. The main chemicals of aqueous extract of *S. miltiorrhiza* are caffeic acid metabolites (Lu and Foo, 2002; Wang, 2010). Some of the pharmacological activities of *S. miltiorrhiza* in traditional medicine are protection of myocardium derangement induced by ischemia, protection of neural cells injuries induced by anoxia, inhibition of the platelet aggregation, hepatic fibrosis and HIV-1 (Lay et al., 2003). Also, this plant is used as a protective agent on vascular endothelial cells (Wang et al., 2010). It has protective effect on disorders that contributed to endothelial dysfunction or vascular endothelial injury, like focal cerebral infarction and cerebral ischemia (Cai et al., 2007).

Wang Y. et al. (2015) suggested that treatment of pulmonary hypertensive rats with *S. miltiorrhiza* aqueous extract could improve hemodynamic parameters such as mPAP and RVSP. It also increased NO and 6-Keto-PGF1*α* and decreased ET-1 and TXB2 levels in the plasma and down-regulated the expression of TGF-β1 in lung tissue which is one of the important factors in inducing vascular remodeling and consequently, PH progression. Endothelial injury causes imbalance of vasoactive substances such as vasorelaxant (PGI2 and NO), and vasoconstrictors (ET-1 and TXA2). These results suggest that *S. miltiorrhiza* can be considered as a treatment for PH by increasing NO levels and decreasing ET-1, TXA2, and TGF-β1 expression in lung tissue and improvement of hemodynamic indices.

Si-chuan et al. (1994) in a study on hypoxia induced pulmonary hypertensive rats suggested that *S. miltiorrhiza* can be an effective treatment for PH due to its reducing activity on RVSP and RVHI. Chen et al. (2003) demonstrated that *S. miltiorrhiza* in pulmonary hypertensive rats could reduce thickening in the SPAs, could increase the levels of HO-1 and iNOS and decrease the expression of eNOS. In high altitude conditions, HO-CO pathway is up-regulated and the expression of HO-1 protein and the CO production increased. In other words, HO-CO is a pathway related to pulmonary arterial vasodilation and protective from pathological remodeling (Llanos et al., 2012). Therefore, according to the available evidence, *S. miltiorrhiza* could ameliorate PH.

##### Securigera securidaca *(L.) Degen & Dörfl*.

*Securigera securidaca* (L.) Degen & Dörfl. (Goat pea), a member of Fabaceae family, that grows at West Asia, Europe and Africa. It is used in traditional medicine for treatment of many diseases such as chronic obesity, diabetes, central nervous system diseases, high blood pressure, and digestive problems (Hosseinzadeh et al., 2002). Flavonoids, alkaloids and saponins are found in its aqueous and alcoholic extracts (Ahmadi et al., 2016). Ahmadipour (2018) reported that the treatment with *S. securidaca* in broiler chickens with PH could increase NO levels in plasma. NO is a vasorelaxant agent and can improve PH. The flavonoids found in *S. securidaca* could protect NO from inactivation by scavenging superoxide anions. The results of Ahmadipour's study show that the treatment with *S. securidaca* could improve hemodynamic parameters and can decrease R, S and T wave amplitudes of the electrocardiogram. This plant can reduce oxidative stress by reducing MDA levels. It has been suggested that the antioxidant properties of *S. securidaca* are due to phytosterols, 4‐Methyl‐2,6‐di‐tertbutylphenol, palmitic acid, 9,12-octadecadienoic acid and alkaloidal compounds. Therefore, these compounds could be considered as an appropriate treatment for PH (Ahmadipour, 2018). Several studies demonstrated that there is a strong relationship between ET-1 expression and PH. ET-1 expression in PH broilers chicken increased compared with normal broilers (Gómez et al., 2007). Studies show that *S. securidaca* can reduce ET-1 expression in high altitude-induced PH in broiler chicken (Ahmadipour, 2018). Also, flavonoids found in *S. securidaca* can reduce transmembrane 45Ca^2+^ absorption or inhibit protein kinase C activity, inhibit cAMP‐ and cGMP-phosphodiesterase (PDE) (Beretz et al., 1982) and that eventually leads to reduction in contraction of the vascular system and PH improvement. Therefore, *S. securidaca* with its vasorelaxant and antioxidant activities can ameliorate outcomes of PH (Ahmadipour, 2018).

##### Terminalia arjuna *(Roxb. ex DC.) Wight & Arn.*

*Terminalia arjuna* (Roxb. ex DC.) Wight & Arn. (Arjuna) is an evergreen tree grow in India, a member of the Combretaceae family. It is used in traditional medicine to improve cardiovascular disease (Dwivedi, 2007). Some of the active compounds of *T. arjuna* include flavonoids, terpenoids, tannins and minerals (Varghese et al., 2015). Some of the pharmacological activities of *T. arjuna* are anti-ischemic, antioxidant, hypolipidemi, increasing cardiac contraction and decreasing blood pressure, (Pawar and Bhutani, 2005; Kapoor et al., 2014).

Meghwani et al. (2017) found that the aqueous extract of *T. arjuna* stem bark can prevent the decrease of relative weight of lungs in pulmonary hypertensive rats. The study was performed on MCT-induced PH rats. The effects of *T. arjuna* are associated also with reduction of RV hypertrophy and %MWT of pulmonary artery, with decrease lipid peroxidation and NOX1 protein expression in lung and with increase the SOD and CAT, also with decrease in BCL2/BAX ratio mRNA demonstrating antiapoptotic effect of the *T. arjuna* in pulmonary hypertensive rats. The results of these studies suggest that *T. arjuna* can be considered as an effective treatment for PH.

##### Trifolium pratense *L.*

*Trifolium pratense* L. (Red clover) belongs to Fabaceae family. It is native to Europe, West Asia and Northwest Africa. Several types of flavonoids are essential constituents of *T. pratense* (Boué et al., 2003). A study on animal models suggested that isoflavones in *T. pratense* can reduce contractions of the smooth muscle in ileum, uterus and bladder (Wang et al., 2008). Jiang and Yang (2016) demonstrated that the use of isoflavones of *T. pratense* extract, mixed in food, in broiler chickens with PH could reduce ET-1 in serum and lungs and could increase the secretion of NOS. ET-1 can increase the MMP-2 expression that is related to pulmonary vascular remodeling (Tan et al., 2012). *T. pratense* has phytoestrogen activity and can increase NOS and NO levels in the serum (Simoncini et al., 2005). All of these findings suggested that *T. pratense* isoflavones have therapeutic potential for PH in bird models by increasing in NO and decreasing in ET-1 expression.

##### Withania somnifera *(L.) Dunal*

*Withania somnifera* (L.) Dunal (Ashwagandha) is a herbal remedy belongs to Solanaceae family which is used for treatment of many diseases especially in India (Mohanty et al., 2004). HPLC analysis shows withaferin A and withanolide A as the most important active components of *W. somnifera* root powder extract (Kaur et al., 2015). Pharmacological uses of *W. somnifera* include anti-fatigue, bone enhancer, anti-aging, anti-Alzheimer, anti-inflammatory, antioxidant, hypoglycemic and anticancer (Ojha and Arya, 2009). It is used also to lower blood pressure and to lower cholesterol levels in the blood (Mohanty et al., 2008).

Kaur et al. (2015) during a study on MCT induced pulmonary hypertensive rats shows that the treatment with *W. somnifera* can reduce RVP and RVH, also could reduce the expression of PCNA. The treatment with *W. somnifera* causes an increase in procaspase-3 expression, and therefore inducement of apoptosis in pulmonary vessels. In addition, treatment with *W. somnifera* could reduce ROS levels in lung tissue. It can increase the levels of IL-10 and decrease in levels of TNF-α and NFkB, which is in connection with its anti-inflammatory effect. Withanolides found that *W. somnifera* inhibit the activation of NFkB by inhibition of TNF-α and show the anti-inflammatory effect of *W. somnifera* (Ichikawa et al., 2006). *W. somnifera* also increases eNOS expression and decreases HIF-1α expression in lung tissue (HIF-1α increases in hypoxia conditions). Increased eNOS activity can increase NO levels, which cause vasorelaxation (Kaur et al., 2015). According to the mentioned studies, *W. somnifera* could be a potential herb in the treatment of PH by its antioxidant, vasorelaxant and anti-inflammatory activities.

#### Phytochemicals Used for PH Treatment

##### Apple Polyphenol

Apple polyphenol is a compound that derived from different types of apple fruits (*Malus* spp.), with many pharmacological activities for treatment of diseases such as gastric mucosal damage, cancers, inflammatory and cardiovascular diseases (Espley et al., 2013).

Hua et al. (2018) during a study on hypoxia-induced PH in rats found that treatment with apple polyphenol can modulate hemodynamic indicators such as mPAP and PVR and therefore could decrease the contraction of pulmonary vessel rings. *In vitro* it decreases cytosolic Ca^2+^ in PASMCs. At the genetic level, apple polyphenol causes an increase of eNOS expression, an increase of NO levels and decrease in caspase-3 and iNOS expression in PAEC. Therefore, all of these findings confirm that apple polyphenol can be considered as a treatment for PH that induces its effect by reducing of contraction and increasing of NO levels.

##### Asiaticoside

Asiaticoside is a saponin derived from *Centella asiatica* (L.) Urb. and it has many applications in traditional medicine with high range of biological activities such as antioxidant, anti-inflammatory, anti-hepatofibrotic (Dong et al., 2004) and neuroprotection effect on transient cerebral ischemia and reperfusion (Chen et al., 2014).

Wang et al. (2018) during a study on hypoxia induced PH shows that treatment with asiaticoside decreased the RVH and mPAP, also increase the concentration of cGMP and NO and decrease circulating concentration of ET-1. Additionally, this compound increase the activity of AKT, the phosphorylation and therefore leads to activation of eNOS and to inhibition of endothelial cells apoptosis. Studies suggested that asiaticoside can enhance phosphorylation and activation of serine/threonine-proteinn kinase/eNOS pathway and therefore to increase NO production that inhibits the progression of PH.

Wang X. B. et al. (2015) during an *in vivo* and *in vitro* investigation on hypoxia induced PH in rats and PASMCs demonstrated that treatment with asiaticoside improves the mPAP and RVH, also reduces the expression of TGF-β1, TGF-βR1, and TGF-βR2 and inhibits the phosphorylation of Smad2/3 in lung tissue of animals. It has been shown that Smad2/3 phosphorylation raises during PH progression (Richter et al., 2004). In PASMCs asiaticoside reduces the cell number and migration, also reduces the TGF-β1 expression. So asiaticoside do its therapeutic effect by reducing proliferation and raising apoptosis (Wang X. B. et al., 2015).

##### Astragalus Polysaccharides

*Astragalus* polysaccharides are active compounds derived from *Astragalus membranaceus* Fisch. ex Bunge, that have many pharmacological activities especially anti-inflammatory and antioxidant (Auyeung et al., 2016).

Yuan et al. (2017) during a study on MCT induced PH in rats demonstrated that treatment with *Astragalus* polysaccharides causes improvement in hemodynamic indicators such as decreasing mPAP, PVR, RVH and medial wall thickness. In addition, overexpression of eNOS and increase of NO levels were observed in treatment groups. Decrease in pro-inflammatory mediators levels were observed due to treatment with *Astragalus* polysaccharides. Also, decrease in the expression of pho-IκBα was confirmed. Pho-IκBα is a marker for assessment of inflammation, so according to the studies *Astragalus* polysaccharides can be a useful treatment for PH by inhibition of the inflammation and by increasing of NO levels leading to improvement of hemodynamic indicators.

##### Baicalin

Baicalin is a flavonoid derived from *Scutellaria baicalensis* Georgi roots and it is used in traditional medicine with many pharmacological activities such as antioxidant, anticancer, anti-inflammatory and inducing apoptosis (Dong et al., 2010). Studies show that baicalin could inhibit the expression of collagen I due to its anti-fibrotic activity (Hu et al., 2009). Wang et al. (2013) demonstrated that pulmonary arterial collagen is an important factor in PH due to hypoxic conditions.

Liu et al. (2015) during a study on hypoxia-induced PH shows that treatment with baicalin cause improvement of hemodynamic factors including mPAP, mSAP, and RV/(LV+S)%. Also, baicalin reduces protein and mRNA expression of collagen I in lung tissue, which is an index for PH progression. It should be noted that baicalin has small effect on collagen III protein and mRNA expression compared with its great effect on collagen I mRNA and protein expression. Baicalin increases the expression of ADAMTS1 and therefore inhibits the expression of collagen I. Therefore, baicain can be considered as a treatment for PH due to its inhibitory effect on collagen I synthesis and improvement in hemodynamic factors (Liu et al., 2015).

Huang et al. (2014), during a study on HPASMCs exposed to TGF-β1, demonstrated that treatment with baicalin decrease the expression of HIF-1α and AhR that are responsible for cell proliferation. So treatment with baicalin, *in vitro*, cause decrease of proliferation and it can be considered for improving the conditions caused by PH. Studied prove that treatment with baicalin can improve PH by anti-proliferation effect mediated by the AhR pathway.

Hsu et al. (2018) during a study on MCT induced PH in rats shows that treatment with baicalin causes improvement of hemodynamic indicators such as RVSP and RVH, also reduction of ET-1 and ETA (ETAR) protein receptor expression in lungs and therefore leads to suppression of the Akt/ERK1/2/GSK3β/β catenin signaling pathway. Activation of this pathway cause increase of the ET-1 expression and progression of PH. Additionally, the reduction of superoxide production and increase the expression of eNOS were observed in treated rats. It has been demonstrated that the treatment with baicalin causes decrease of von Willebrand factor (vWF), which is a marker of endothelial injury (Hsu et al., 2018). These results suggest that baicalin can be considered as a potent treatment for PH.

##### Berberine

Berberine is an isoquinoline alkaloid derived from some Chinese herbs, such as *Coptidis* rhizome (Zhu and Qian, 2006). It has many pharmacological activities: antimicrobial, antiproliferative, cardioprotective, antidiabetic, anticancer and others (Gao et al., 2017; Neag et al., 2018; Belwal et al., 2020).

Luo et al. (2018) during a study shows that treatment with berberine cause decrease of protein phosphatase 2A (PP2Ac) phosphorylation, which has an important role in the progression of PH. Therefore the decrease of PP2Ac expression cause decrease in the proliferation and migration of PASMCs which leads to improvement of PH; also treatment with berberine leads to improvement in hemodynamic indicators such as RV/(LV + S) and to reduction of medial wall thickness percentage.

##### Carvacrol

Carvacrol is a monoterpenoid phenol, derived from oregano (*Origanum* spp.) and thyme (*Thymus* spp.) leaves. Some of the most important pharmacological activities of carvacrol are antibacterial, antiviral, antioxidant and anticancer (Sökmen et al., 2004).

Zhang et al. (2016) during a study on hypoxia-induced PH in rats demonstrated that carvacrol reduces RVH and the thickening of pulmonary arteries. Also, it can reduce oxidative stress by increasing SOD and GSH activity and decreasing MDA levels in PASMCs. Treatment with carvacrol reduces the levels of mRNA and protein expression of Bcl-2 (anti-apoptotic protein) and reduces the Procaspase-3 expression, raises the caspase-3 expression and so it can increase PASMCs apoptosis. Also, ERK1/2 and Akt increase cell survival and decrease apoptosis. Additionally, carvacrol treatment inhibits the ERK1/2 and PI3K/Akt pathway. All of these findings demonstrate that carvacrol can ameliorate PH by increasing apoptosis and decreasing oxidative stress in PASMCs.

##### Genistein

Genistein is a phytoestrogen which has higher affinity to ERβ compared with ERα. This natural compound has many pharmacological activities such as vasodilator, cardioprotective, and anti-inflammatory. It has been suggested that genistein has protective effect on PH progression (Homma et al., 2006).

Matori et al. (2012) during a study on MCT induced PH shows that treatment with genistein can improve hemodynamic indicators including RVP, RVH, RVEF, and RV dilatation. Genistein also decreases pulmonary fibrosis and tissue injuries due to PH. Genistein causes increase in protein expression of ERβ in lung and RV. *In vitro* studies show that genistein inhibits the proliferation of HPASMCs and hypertrophy of NRVM.

All of the protective effects of genistein on PH are related to its phytoestrogenic activity. Various studies have shown that estrogens have protective effects against cardiovascular and PH disease and this effect is *via* estrogen receptor-β (ERβ) (Umar et al., 2011). Therefore, genistein as a phytoestrogen, can modulate PH outcomes by affecting on ERβ expression.

##### Ginsenoside Rb1

Ginsenoside Rb1 is one of the phytochemicals derived from *Panax ginseng* that shows many pharmacological activities especially in the treatment of heart and lung diseases like treatment of endothelial injuries and inflammation disorders in endothelial smooth muscle cells (Yu et al., 2007; Li et al., 2011).

Wang R. X. et al. (2015) during an investigation on hypoxia and MCT induced PH in rats demonstrated that treatment with Ginsenoside Rb1 improves PH *in vivo* and *in vitro* by improving RV/(LV+S) ratio, reducing the expression of ET-1 and SOCE and induces relaxation in PAs. The concentration of cytosolic Ca^2+^ adjusted by SOCE in PASMCs and it's a key factor for contraction and proliferation of PASMCs. Ginsenoside Rb1 do its therapeutic effect by reducing the ET-1 and SOCE expression and reducing contraction, so it can be considered as a treatment for PH.

##### Isorhynchophylline

Isorhynchophylline is an alkaloid derived from *Uncaria rhynchophylla* (Miq.) Miq. ex Havil. and it is widely used in traditional medicine especially for treatment of heart and brain diseases (Zhou and Zhou, 2012). Some of the pharmacological activities of isorhynchophylline include reducing oxidative stress, apoptosis, anticancer and anti-inflammatory (Kawakami et al., 2011).

Guo et al. (2014) during a study on MCT induced PH in rats shows that treatment with isorhynchophylline can improve RVP and RVH. Also, isorhynchophylline can reduce PASMCs proliferation *in vitro*. They also demonstrated that isorhynchophylline can reduce the expression of Cyclin D1 and CDK6, and can increase the expression of p27Kip1 and therefore it can regulate cell cycle and could reduce proliferation. Isorhynchophylline inhibits ERK1/2 and STAT3 signaling pathways and this way it can suppress cell growth (Guo et al., 2014). All of these findings suggested that isorhynchophylline can be considered as a potent treatment for PH that causes regulation of cell cycle and inhibit cell growth and proliferation.

##### Magnesium lithospermate B

Magnesium lithospermate B is a phytochemical isolated from *Salvia miltiorrhiza*. Studies shows that magnesium lithospermate B is a potent antioxidant that do its effect through inhibition of NADPH oxidase; NADPH oxidase (NOX) is a factor contributing to increased ROS and cell damage (Lou et al., 2015; Jin et al., 2016).

Li et al. (2019) during an *in vivo* and *in vitro* investigation shows that treatment of pulmonary hypertensive rats and PASMCs with magnesium lithospermate B reduces the expression of NOX2 and NOX4 and ERK in PAs, also reduces the ROS and H_2_O_2_ levels. In PASMCs, it reduces OPN and Cyclin D1 and raises α-SMA and SM22α expression (contractile genes with high and low rate of proliferation, respectively). So, based on these findings magnesium lithospermate B can be considered as a potent treatment of PH by modulating oxidative stress in PAs of animals and modulating contractile genes in PASMCs.

##### Nobiletin

Nobiletin is a flavonoid derived from peels of citrus fruits. This compound has many applications due to its biological activities such as anti-inflammatory, antioxidant and anti-tumor (Huang et al., 2016).

Cheng et al. (2017) during a study on MCT induced pulmonary hypertensive rats suggested that treatment with nobiletin leads to improvement of hemodynamic indicators such as RVSP and RVH and therefore has a positive effect on pulmonary vascular remodeling induced by hypoxia. In *in vitro* conditions, nobiletin suppresses PDGF-BB, Src and STAT3 phosphorylation, after that it suppresses Pim1 and NFATc2, which are target genes of Src and STAT3. Finally, this gene cascade inhibits proliferation. Therefore, nobiletin can prevent the progression of PH by its inhibition effect on Src/STAT3 axis that is one of the pathways of proliferation.

##### Oxymatrine

Oxymatrine is a phytochemical found in *Sophora flavescens* Ait. that shows many pharmacological activities such as anti-inflammatory and antioxidant activities especially in lung and heart tissues, compared to other organs (Jun-xue and Guo-jun, 2000); studies shows its protective effect against lung injury (Xu et al., 2005).

Zhang et al. (2014) during an investigation on hypoxia and MCT induced PH in rats (*in vivo*) and PASMCs (*in vitro*) shows that it cause improvement of PH by reducing the mRNA expression of inflammatory factors, chemokines and cytokines, growth and adhesion factors including MCP-1, IL-6, SDF-1, TGF-β, VEGF, ICAM-1, and VCAM-1. It also improves hemodynamic indicators. In addition, oxymatrine decreases the expression of HIF-1α and p-NF-κB in lung tissue and increased antioxidant factors including Nrf2, SOD, Ho-1 and GSH in PASMCs. This phytochemical can modulate PH *in vitro* and *in vivo* and in both hypoxia and MCT models by its antioxidant, antiproliferative and antiinflammatory effects.

##### Panax notoginseng *Saponins*

*Panax notoginseng* Saponins (PNS) are compounds that are used in traditional Chinese medicine due to their biological and therapeutic activities such as vasorelaxant, antioxidant and inhibitory effect on vascular smooth muscle receptor of the voltage-gated Ca^2+^ channels (Chu et al., 2007).

Zhao et al. (2015) during a study on PNS injection on pulmonary hypertensive rats induced by hypoxia, shows that PNS can improve hemodynamic indicators such as mPAP, mCAP and RV/(LV+S) ratio and therefore can reduce the expression of p38MAPK mRNA in lung tissue of pulmonary hypertensive rats. P38MAPK is an important index for physiological stress and its expression increases during PH progression. So according to the studies, PNS could be considered as a treatment for PH.

##### Polydatin

Polydatin is a phytochemical derived from *Polygonum cuspidatum* and has many pharmacological and therapeutic activities such as antiinflammatory, antioxidant, anticancer and inhibition of platelet aggregation (Miao et al., 2011).

Studies shows that during the progression of PH, increase in the levels of endothelin and angiotensin II leads to reduce in the bioavailability of NO (Berger et al., 2009). Miao et al. (2012) during an investigation on hypoxia induced PH in rats showed that treatment with polydatin increases the NO levels *via* reduction of endothelin and angiotensin II levels in lung and serum. It also cause improvement of hemodynamic indicators like mPAP, mCAP and RVH.

##### Punicalagin

Punicalagin is a phytochemical isolated from pomegranate juice and has many pharmacological activities due to its high antioxidant content (Xu et al., 2015); also it has been reported that punicalagin used for treatment of lung diseases in animal models (Peng et al., 2015).

Shao et al. (2016) during an investigation on hypoxia induced PH in rats shows that treatment with punicalagin improves hemodynamic indicators and induce relaxation by enhancing NO-cGMP signaling pathway in PAs. Also this phytochemical reduces oxidative stress and raises mRNA expression of MMP-9, HIF-1α, VEGFA, NF-kB and TNF-α in lung tissue of animals. All of these factors increase in hypoxia conditions and as mentioned, they returned to their normal level by punicalagin treatment. So this phytochemical can be considered as an effective treatment for PH.

##### Quercetin

Quercetin is a flavonoid found in a wide range of plants and fruits with many biological activities such as antioxidant, anti-inflammatory, antitumor, antiproliferative, inducing apoptosis, antimetastatic, vasodilator and decreasing blood pressure (Muhammad et al., 2015). Also, it has been suggested that quercetin inhibits the progression of PH in rats (Morales-Cano et al., 2014).

He et al. (2015) during a study on hypoxia induced PH in rats demonstrated that treatment with quercetin improves hemodynamic indicators such as PAH and RVH. Quercetin can inhibit the proliferation of PASMCs and can induce apoptosis in cells by inhibiting the TrkA/AKT signaling pathway. It was confirmed that quercetin increases the BAX expression and decreases the expression of Bcl-2. Additionally, quercetin causes an increase of cyclin D1 and a decrease of cyclin B1 and Cdc2 protein expression. They are responsible for cell cycle regulation and reduce the expression of MMP2, MMP9, CXCR4, integrin β1 and integrin α5 which are responsible for cell migration. Gao, H. et al (2012) during an investigation on MCT induced pulmonary hypertensive rats demonstrated that treatment with quercetin reduces the proliferation in PASMCs *via* reducing the expression of PCNA gene also quercetin reduces mPAP and RVH and WT in treated rats. So they shows that quercetin do its therapeutic effect by inhibition of proliferation in PASMCs. Therefore, quercetin can be considered as a treatment for PH by its prevention effect on migration and by its apoptosis inducing effect.

##### Resveratrol

Resveratrol is a powerful antioxidant with a polyphenolic structure. It has many biological activities and therefore has many therapeutic applications such as antiinflammatory, antioxidant and antiaging (Ji et al., 2013; Yeung et al., 2019). The anticancer activity of resveratrol is due to its inhibition of ACT, which leads to increase of apoptosis in human uterine cancer cells (Testa and Bellacosa, 2001).

Guan et al. (2017) during a study on pulmonary artery vascular smooth muscle cells (PASMCs) which have been exposed to hypoxia, demonstrated that treatment with resveratrol could inhibit proliferation and migration of PASMCs and after that could reduce AKT and p-AKT expression in PAMSCs. Also, resveratrol decreases the expression of MMP-2 and MMP-9. MMPs are responsible for increased migration and proliferation of PASMCs by activating the PI3K/AKT signaling pathway (Shivakrupa et al., 2003).

It has been suggested that resveratrol can increase the expression of *NO*, decrease inflammation, cell proliferation and oxidative stress. Therefore, it could improve the function of pulmonary artery endothelium and PH. Additionally, resveratrol can inhibit dysfunctions of rat pulmonary artery vessels (like inflammation and contraction that leads to PAH) and cardiomyocyte hypertrophy (Paffett et al., 2012).

Xu et al. (2016) during a study on hypoxia-induced PH *in vivo* and *in vitro*, demonstrated that resveratrol could improve PH and could reduce inflammatory mediators in the lungs of rats. Also treatments with resveratrol cause decrease of H_2_O_2_ in rats lungs. HIF-1α is an important gene in PH progression, NO and ROS activate HIF-1α expression (Prabhakar and Semenza, 2012) and resveratrol decreases its expression by its antioxidant activity. Trx-1 and Nrf-2 proteins expression were increased by resveratrol. Nrf2 is an antioxidant marker that has balancing effects on antioxidant proteins like Trx-1. Resveratrol increases GSH and SOD activities, Trx-1 and Nrf-2 protein levels in PASMCs and it decreases the proliferation of PASMCs and ROS, H_2_O_2_ production and expression of HIF-1α. All of these markers shows that resveratrol is a powerful antioxidant (Xu et al., 2016).

Trimethoxystilbene (TMS) is a synthesized compound from methylation of resveratrol that has more activities and low toxicity compared with resveratrol (Ma et al., 2009). Gao, G., et al (2012) during an *in vitro* study on PASMCs shows that TMS is more potent in treatment of PH compared to resveratrol, by increasing induction of apoptosis and decreasing cell proliferation. Therefore, it is suggested that resveratrol can be a potent treatment for PH due to its antioxidant and anti-proliferative activities.

##### Rhoifolin, Apiin and Luteolin

Rhoifolin, apiin and luteolin are three types of flavonoids used in traditional medicine. Studies show that flavonoids are present in the majority of vegetables and plants. Some of the biological and pharmacological activities of these flavonoids are anti-inflammatory, antiallergic and spasmolytic (Gabor, 1986). It has been suggested that luteolin, apiin and rhoifolin have inhibitory effects on slow fibers of the dog heart; also they can reduce aortic pressure and capillary pulmonary pressure in normal animals (Occhiuto et al., 1988).

Occhiuto and Limardi (1994), during a study on hypoxia-induced PH in dogs, demonstrated that structural changes of flavonoids (luteolin, apiin and rhoifolin) cause significant changes in antihypertensive activity. They showed that rhoifolin reduces aortic pressure but has not effect on pulmonary vascular resistance while luteolin and apiin have antihypertensive properties.

##### Salidroside

Salidroside is a phytochemical isolated from *Rhodiola rosea* L. that widely used for PH treatment and has many pharmacological applications such as antioxidant, anticancer, antiinflammation and immune system enhancer (Panossian and Wagner, 2005; Guan et al., 2011).

Huang et al. (2015) during an investigation on pulmonary hypertensive mice demonstrated that treatment with salidroside reduces RVH in treated mice and raises Caspase-3, BAX, A_2a_R and reduces BCL2 expression in PASMCs. Adenosine A_2a_R has protective effect in PH progression and inhibits vascular remodeling process. So according to this study, salidroside can be considered as a cure for PH by regulating apoptosis pathway.

## Discussion

PH is a progressive disease characterized by increase in pulmonary blood pressure, right ventricular hypertrophy and thickening of the vascular intima, media, and adventitia (Voelkel et al., 2012). Vascular thickening is due to increased proliferation and resistant to apoptosis in PASMCs (Xu et al., 2016). Many signaling pathways are responsible in this regard such as Ca^2+^ signaling pathway (Reyes et al., 2018); JAK/STAT (Wang et al., 2005); RhoA/ROCKs (Antoniu, 2012); Notch (Yamamura et al., 2014), and Src/STAT3 (Paulin et al., 2011).

Despite recent advances in medicine and pharmacy, industrial remedies are not sufficient to prevent the progression of the PH (Lythgoe et al., 2016). Therefore, introduction and evaluation of natural compounds effective in the treatment of PH seems necessary.

According to our study, chemical compounds that are effective in treatment of PH include flavonoids, isoflavonoids, monoterpenoid phenolics DMDS, polymethoxylated flavones, terpenoids, phytoesterogens, isoquinoline alkaloids, saponins, polysaccharides, tetracyclic oxindole alkaloid.

**Figure 2:**
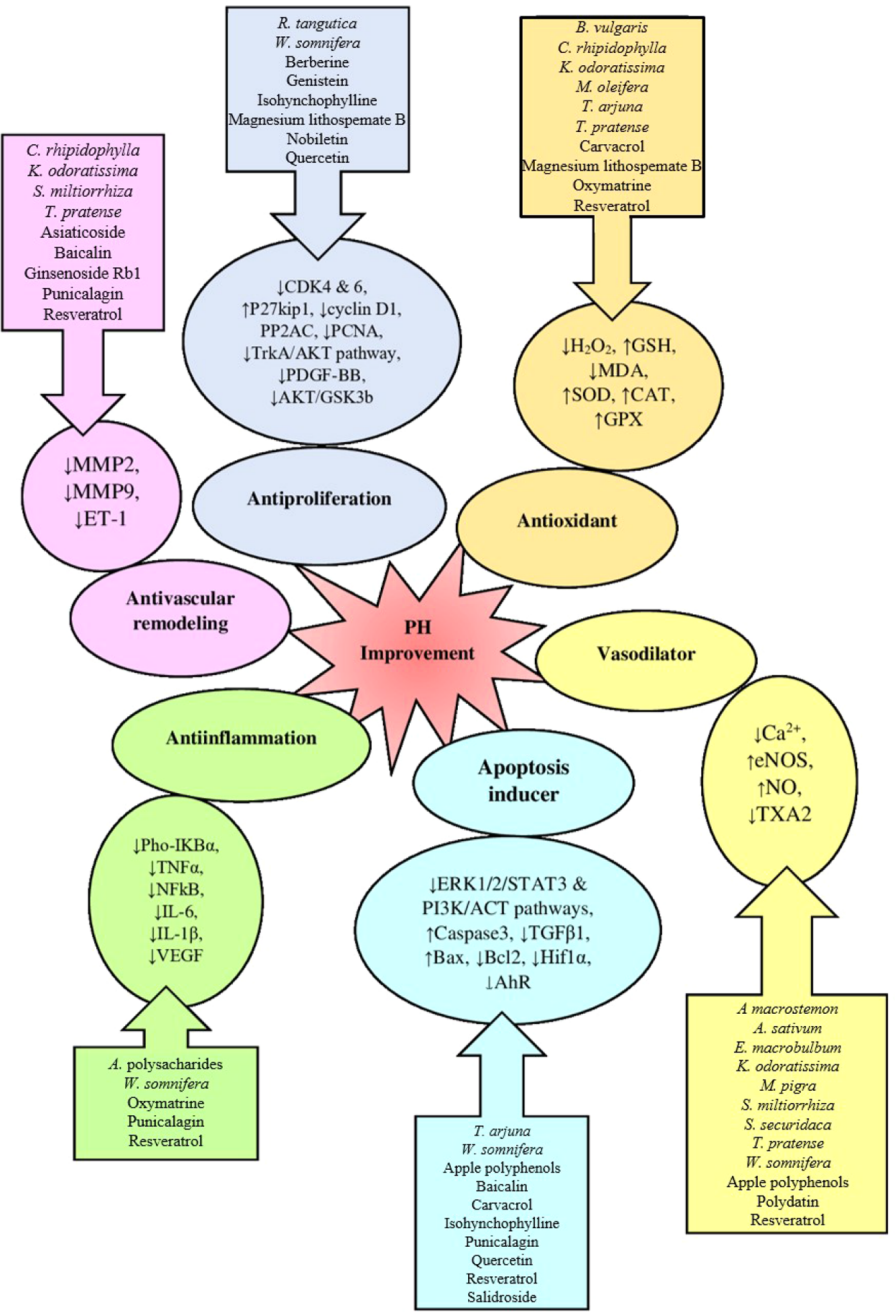
The summary of 6 pathways that leads to PH improvement.

All herbs and phytochemicals induce their therapeutic effect through one of the 6 pathways (**Figure 2**):

- **Antiproliferation:** Plant extracts and phytochemicals that have antiproliferative effects include *R. tangutica, W. somnifera,* berberine, genistein, isohynchophylline, magnesium lithospemate B, nobiletin, and quercetin, which do their effects by reducing expression or suppressing the pathways involved in cell proliferation. Some of these markers and pathways decreases the expression of cyclin D1, CDK 4 and 6, PP2AC, PDGF-BB, and PCNA markers and increases the p27kip1 (cyclin inhibitor) marker, that suppresses Trk A/ACT and ACT/GSK3b pathways; the final result is reduction of proliferation (Guo et al., 2014; He et al., 2015; Kaur et al., 2015; Cheng et al., 2017; Luo et al., 2018; Nan et al., 2018).
- **Antioxidant:** Plant extracts and phytochemicals with antioxidant properties on treatment of PH include *B. vulgaris, C. rhipidophylla, K. odoratissima, M. oleifera, T. arjuna, T. pratense,* carvacrol, magnesium lithospemate B, oxymatrine and resveratrol, which do their effect by increasing CAT, SOD, GPx, GSH levels and reducing H_2_O_2_, ROS, and MDA levels. All of these indicators show decrease oxidative stress and reduce tissue damage (Chen et al., 2012; Ahmadipour et al., 2015; Kaur et al., 2015; Jiang and Yang, 2016; Xu et al., 2016; Zhang et al., 2016; Guan et al., 2017; Meghwani et al., 2017; Ahmadipour et al., 2019).
- **Antivascular remodeling:** Vascular remodeling is one of the important events during PH progression that has been explained before. Many plant extracts and phytochemicals do its effects by suppressing ET-1 expression and following that MMP-2 and MMP-9 expression which cause inhibition of the progression of vascular remodeling. Some of these plant extracts and phytochemicals include *C. rhipidophylla, K. odoratissima, S. miltiorrhiza, T. pratense,* asiaticoside, baicalin, ginsenoside Rb1, punicalagin and resveratrol; that have vascular antiremodeling effect and this way inhibit the progression of PH (Chen et al., 2003; Ahmadipour et al., 2015; He et al., 2015; Jiang and Yang, 2016; Xu et al., 2016; Ahmadipour et al., 2017; Guan et al., 2017; Hsu et al., 2018; Wang et al., 2018; Ahmadipour et al., 2019).
- **Vasodilator:** One of the important reasons for PH is increased blood vessel contraction and after that PH. The most important vascular relaxation inducers do their effect by increasing NO levels due to increasing eNOS expression and decreasing the levels of TXA2 and cytosolic Ca^2+^. Plant extracts and phytochemicals with these properties include *A macrostemon, A. sativum, E. macrobulbum, K. odoratissima, M. pigra, S. miltiorrhiza, S. securidaca, T. pratense, W. somnifera,* Apple polyphenols, polydatin and resveratrol (Fallon et al., 1998; Chen et al., 2003; Rakotomalala et al., 2013; Kaur et al., 2015; Ahmadipour et al., 2015; Jiang and Yang, 2016; Han et al., 2017; Hua et al., 2018).
- **Apoptosis inducer:** Some of the plant extracts and phytochemicals cause improvement in PH by increasing apoptosis such as *T. arjuna, W. somnifera, apple polyphenols,* baicalin, carvacrol, isohynchophylline, punicalagin, quercetin, resveratrol and salidroside. They act by inhibiting of growth factors such as TGF-β1, AhR and HIF-1α or suppressing PI3K/ACT, ERK1/2/STAT3 pathways, or increase the expression of BAX and caspase-3 (apoptosis inducers) and decrease the expression of BCL2 (anti-apoptosis factor). All of these agents drive the cells (lung tissue cells, PAECs and PASMCs) toward apoptosis and inhibit the progression of PH (Guo et al., 2014; Huang et al., 2014; Kaur et al., 2015; Wang Y. et al., 2015; He et al., 2015; Zhang et al., 2016; Guan et al., 2017; Meghwani et al., 2017; Hua et al., 2018).
- **Anti-inflammation:** One of the notable processes in PH is increased inflammation. Some of the plant extracts and phytochemicals such as *Astragalus polysacharides, W. somnifera*, oxymatrine, punicalagin and resveratrol, can reduce this inflammation by decreasing inflammation factors such as IL-10, IL-6, IL-1β, TNF-α, NFkB, VEGF, pho-IkBα. Therefore, they can inhibit inflammation and following that can reduce the progression of PH (Kaur et al., 2015; Xu et al., 2016; Yuan et al., 2017).

## Future Direction

The present study provides comprehensive information about medicinal plants and phytochemicals used for PH treatment, as discussed, all these herbs and phytochemicals have therapeutic effects on PH but exert their effects in different ways. There are still unknown mechanisms to industrialization of these medicinal plants and phytochemicals including purification of active compounds of herbs, combining several herbs or phytochemicals to improve their effectiveness (in this case, pharmacological activities, toxicity and herb/food/drug interaction should be completely investigated). Bioinformatics studies are suggested to determine the interaction between active compounds or phytochemicals and their target receptors or target cells. Further studies to identify the signaling pathways are suggested. Making suitable formulations with optimal bioavailability and clinical trials to evaluating the effect of herbs and phytochemicals on patients suffering from PH and investigating the side effects, toxicity, safely and effectiveness of these treatments can provide the field for use in pharmacy.

## Conclusion

According to this review study, as described, many herbs and natural compounds have the potential for adjuvant therapy of PH treatment without side effects of chemical drugs. In addition, as mentioned, there is no definitive cure for PH, so use of herbs and phytochemicals can help to improve the quality of life in patients with this disorder.

## Author Contributions

MF and SJ designed the study and collected the information. JE extracted articles and HK wrote the article. IA and JE revised the text.

## Conflict of Interest

The authors declare that the research was conducted in the absence of any commercial or financial relationships that could be construed as a potential conflict of interest.

## Appendix

**Table:**

5-LOX	5-lipoxygenase
6-Keto-PGF1α	6-keto prostaglandin F1α
A_2a_R	adenosine A_2a_ receptor
α-SMA	α-smooth muscle actin
ADAMTS-1	a disintegrin and metalloprotease with thrombospondin motif type I
Ang II	angiotensin II
AhR	aryl hydrocarbon receptor
Akt	protein kinase B
ALK-I	activin receptor like kinase 1
ALP	alkaline phosphatase
ALT	alanine amino transferase
AST	aspartate amino transferase
BAX	BCL2-associated X protein
BCL2	B-cell lymphoma-2
BMPR2	Bone morphogenetic protein type 2 receptor
BW	body weight
CAT	catalase
CAV-I	Caveolin-1
Cdc2	Cell division control protein 2 homolog 1
CDK4	cyclin protein kinase 4
CDK6	cyclin protein kinase 6
cGMP	Guanosine 3′,5′ cyclic monophosphate
CTEPH	chronic thromboembolic pulmonary hypertension
CXCR4	CXC chemokine receptor 4
DMDS	Dimethyl disulfide
ECG	electrocardiogram
ENG	endoglin
eNOS	endothelial nitric oxide synthase
ERα	human estrogen receptor sub-type β
ERβ	human estrogen receptor sub-type β
ERK1/2	extracellular signal-regulated protein kinases 1 and 2
ET	endothelin
ET-1	endothelin-1
ETAR	endothelin A receptor
ET-1	endothelin-1
GSH	glutathione
GSK3β/β	glycogen synthase kinase 3 beta
GPx	glutathione peroxidase
H/L ratio	heterophil to lymphocyte ratio
HIF-1α	hypoxia-inducible factor 1α
HIV-1	human immunodeficiency virus type 1
HO-1	heme oxygenase 1
HP	Hypoxic pulmonary
HPASMCs	human pulmonary arterial smooth muscle cells
HPVECs	human primary vascular endothelial cells
ICAM-1	intercellular adhesion molecule 1
IL-10	Interleukin 10
IL-1β	Interleukin-1β
IL-6	Interleukin 6
iNOS	inducible nitric oxide synthase
%MWT	percentage medial wall thickness of pulmonary arteries
mCAP	mean carotid artery pressure
MCT	monocrotaline
MDA	malondialdehyde
MMP-2	matrix metalloprotease 2
MMP-9	matrix metalloprotease 9
mCAP	mean carotid arterial pressure
mPAP	mean pulmonary arterial pressure
mSAP	mean systemic arterial pressure
MVW	Medial wall thickness
MWT	medial wall thickness of pulmonary arteries
ND	not detected
NF-κB	nuclear factor κB
NFATc2	calcineurin/nuclear factor of activated T cells c2
NO	nitric oxide
NOX	NADPH oxidases
Nrf 2	nuclear respiratory factor 2
NRVM	neonatal rat ventricular myocyte
OPCs	oligometric proanthocyanidins
OPN	osteopontin
p27KIP1	Cyclin-dependent kinase inhibitor 1B
p38	38.000-dalton membrane protein
p38MAPK	p38 mitogen-activated protein kinase
p-Akt	phosphorylated Akt
PA	pulmonary arterial
PABP	poly-A binding protein
PAEC	pulmonary arterials endothelial cells
PAH	pulmonary arterial hypertension
PAP	pulmonary artery pressure
PASMC	pulmonary arterial smooth muscle cell
PBP	pulmonary blood pressure
PCNA	protein content of proliferating nuclear cell antigen
PDE	phosphodiesterase enzyme
PDE5A	Phosphodiesterase type 5A enzyme
PDGF	platelet derived growth factor
PGI2	prostaglandin I2
PHT	pulmonary hypertension
PI3K	phosphoinositide-3- kinase
PKA	protein kinase A
PKB	protein kinase B
Pim1	provirus integration site for Moloney murine leukemia virus 1
PNS	Panax notoginseng Saponins
PP2Ac	protein phosphatase 2A catalytic subunit (Y307)
PTGS	prostaglandin-endoperoxide synthase
PVC	pulmonary vascular cell
PVR	pulmonary vessel resistance
RES	Resveratrol
RHF	right heart failure
ROS	reactive oxygen species
RV	right ventricle
RVEF	RV ejection fraction
RVH	right ventricular hypertrophy
RVHI	right ventricular hypertrophy index
RVSP	right ventricular systolic pressure
RV/ (LV+S)	ratio of right ventricle to the left ventricular plus interventricular septum
RV/TV ratio	right-to-total ventricular weight ratio
RV/BW	right ventricle to body weight ratio
RVP	right ventricular pressure
S	septum
SMAD3	SMAD Family Member 3
SDF-1	stromal cell-derived factor 1
SOCE	store-operated Ca^2+^ entry
SPA	small pulmonary arteries
SM22α	smooth muscle 22α
SOD-1	superoxide dismutase 1
Src	Proto-Oncogene, Non-Receptor Tyrosine Kinase
STAT3	signal transducer and activator of transcription 3
TAPSE	tricuspid annular plane systolic excursion
TBARS	thiobarbituric acid reactive substance
TGF-β1	transforming growth factor 1
TMS	trimethoxysilbene
TNF-α	Tumor necrosis factor α
TrkA	tropomyosin-related kinase A
Trx-1	thioredoxin reductase 1
TXA2	thromboxane A2
TxB2	thromboxane B2
VCAM-1	vascular cell adhesion molecule 1
VEF	ventricular ejection fraction
VEGF	vascular endothelial growth factor
VEGFA	vascular endothelial growth factor A
vWF	von Willebrand factor
